# Editorial: In Memoriam – Judith Flippen-Anderson (1941–2018)

**DOI:** 10.1063/1.5049131

**Published:** 2018-07-31

**Authors:** Majed Chergui

**Affiliations:** Ecole Polytechnique Fédérale de Lausanne, Laboratory of Ultrafast Spectroscopy (LSU) and Lausanne Centre for Ultrafast Science (LACUS), ISIC, FSB, Station 6, CH-1015 Lausanne, Switzerland

It is with a heavy heart that we learned of the passing of Judith on March 31, 2018. Her role with our journal, *Structural Dynamics*, was integral and valued in a way that words cannot express. She was the key person in the foundation of the relationship between AIP Publishing and the American Crystallographic Association, one of AIP's member societies. A long-time member of the ACA and the crystallography community, Judith spent 35 years as a small molecule crystallographer at the Naval Research Laboratory in Washington, D.C., and later worked for Rutgers University in New Jersey on a project for the Protein Data Bank. She was co-editor of the magazine ACA RefleXions and a former editor of the International Union of Crystallography (IUCr) Newsletter. She was a past chair of the U.S. National Committee for Crystallography, served as ACA president in 1991, and was named to its inaugural class of Fellows in 2011.

Judith served as ACA's representative on the AIP Publishing Board of Managers since its inception in 2013 and was AIP's corporate secretary. In this respect, she was the driving force behind the creation of ACA's first journal, *Structural Dynamics*—an open access title launched jointly with AIP Publishing in 2014. It was during this time where I got to know Judith, as she was the person who interviewed me for the position of Editor-in-Chief on behalf of the ACA. From this first meeting, it was immediately clear that there was accord in our vision for the journal. We understood each other very well, and I was very pleased by how much our views echoed each other. Judith was very supportive of my vision for the journal and accepted the suggestion to name the journal *Structural Dynamics*. Most of all, I remember her constant support and encouragement of strategic initiatives. I was extremely pleased to be accompanied by such a constructive partner in the challenging but fascinating adventure of launching a new journal.

The evolution of the journal was closely followed by Judith, as she was an active member of the team participating in conference calls, attending conferences, and commissioning prominent authors to publish in the journal. In addition, Judith's commitment to promoting the journal was repeatedly showcased in ACA's quarterly newsletter, ACA RefleXions, where featured articles of *Structural Dynamics* were displayed for ACA members. Judith was wonderful in the way she took the destiny of the journal to heart. I recall how excited and happy she was when the journal achieved its first Impact Factor of 3.667 in 2015 and how thrilled she would have been to know the latest Impact Factor of 3.969. The journal and our team owe Judith a debt of gratitude. May she rest in peace.

